# The American Dental Association


**Published:** 1868-10

**Authors:** 


### ARTICLE II.

### *The American Dental Association.* *

The eighth annual meeting of this body was held in Grant Hall, Niagara Falls, in July. The Association is composed of delegates from state and local Dental Societies scattered through the country, but they are at present chiefly located in the Northern States. At the present meeting there were delegations from Maryland, Kentucky, Missouri, Tennessee, and one or two of the other Southern States. The meeting was called to order at 11 o'clock by Dr. A. Lawrence, of Mass., President of the Association. The President congratulated the members present on the largeness of the attendance, the favorable auspices under which they met, and expressed the hope that the time might be passed in a pleasant and profitable manner. The Rev. Mr. Barnes was then introduced, who offered a very feeling and appropriate prayer.

Dr. Whitney in behalf of the Buffalo Dental Association,

---

* Absence from home until the 1st of September, prevented this report from being published in the Sept. No of Journal. We have taken advantage of the excellent report of proceedings by Dr. W. C. Horne, to make the one for the Journal more complete.

made the usual address of welcome to the delegates in attendance, which was received in a very complimentary manner.

On motion of Dr. Shepard, a recess of ten minutes was taken.

After the meeting was called to order, the new members were given an opportunity to sign the constitution.

Resolved, on motion of Dr. Whitney, that the visiting physicians and clergymen present, be invited to take seats in this convention.

The Secretary read the constitution, for the edification of the delegates.

Dr. Geo. B. Snow submitted the report of the Committee on Credentials, which was adopted.

Resolution on receiving delegates from the "American Dental Convention," an organization not entirely dissimilar to this, was partially discussed by Drs. Allport, Rogers, Ambler, J. A. Perkins, and Spellman, and referred to a committee of five to report at the afternoon session.

A committee of nine, according to the provision of the constitution, was appointed for the selection of candidates for the officers of this meeting.

This committee consisted of Drs. H. F. Bishop, W. W. Allport, W. F. Morrill, A. L. Northrup, J. H. McKellops, J. H. McQuillen, L. D. Shepard, W. P. Horton, G. T. Moffatt.

The Association then adjourned until 4 o'clock P. M.

### AFTERNOON SESSION

The meeting was called to order by the President, who requested the Secretary to read the minutes of the last session. After proceding for a short time, the further reading of the minutes was dispensed with.

The committee on credentials made an additional report which was adopted.

Dr. Horne, from the special committee to which was refered the matter of admitting delegates from the American Dental Convention, reported adversely to the admission of said delegates on the ground that said Convention is not a

"local organization." A minority report was made by Dr. Morgan in favor of receiving the delegates from the Convention. After considerable discussion, conducted chiefly by Drs. Rogers, Atkinson, Ambler, J. Allen, McQuillen, Hitchcock, Allport, Whitney, Horne, and others, the resolution offered by the majority of the committee was rejected.

It was then moved that the minority report be adopted, which was passed by a vote of 50 to 44.

The Association then proceeded to ballot for President, when Dr. J. Taft, of Cincinnatti, was elected by a majority of eighteen votes.

Drs. H. Judd, of Missouri, and A. A. Blount, of Ohio, were elected first and second Vice Presidents.

Dr. J. McManus, of Hartford, was chosen Corresponding Secretary.

Dr. Edgar Park, of St. Louis, was chosen Recording Secretary in the place of Dr. Taft, promoted to the Presidency.

Dr. W. H. Goddard was elected Treasurer.

The convention then adjourned till 10 o'clock Wednesday morning.

SECOND DAY'S SESSION.

The meeting was called to order by President Lawrence, who appointed Drs. Horton and Davis to conduct the newly elected President to the chair.

Dr. Taft, on assuming his place, was received in the most complimentary manner. He remarked that he felt duly grateful for their kindness, and was fully aware that the position was one of no little responsibility, and by no means the most pleasant to occupy; but he felt assured that he should have the aid and encouragement of this body. We are engaged, said he, in a great work: let us betake ourselves to the labor before us with willing hands and earnest hearts. His remarks were brief, but well received.

Dr. Edgar Park then took his place as Recording Secretary.

Dr. A. Lawrence, on retiring from the chair, made the following parting address:

*Mr. President and Gentlemen of the American Dental Association*:

According to custom, I suppose I may consider myself called upon to offer a few as appropriate remarks as my abilities will permit on the occasion of leaving the chair to which your kind partiality called me, and in which you have now placed a most worthy successor, and one able to fill it with dignity and honor, both to the Association and to himself.

Permit me to thank you for past favors and predilections, and to request you to give me a willing ear for a few moments while I call your attention to a few such matters as seem specially connected with the interests and prosperities of our Association.

We meet here to day under the most favorable auspices,—harmonious and prosperous. So let our assemblies and relations ever be maintained, to our mutual benefit.

There is no such thing as independence in this universe, and the man who best understands his relationships and dependencies, is in the best condition to enjoy the best of life.

American Dentistry—scarcely two and a half score years of age—has risen from its humble origin and become a highly respectable power for good throughout the country.

At first it was confined to, and practiced by, a few cunning and ingenious workmen, who devised their own plans, instruments and materials, and did the best they could under the circumstances and conditions of their isolations. But such conditons no longer clog and retard us. Now, by concert of movement, by science and association, we have arrived at highly commendable respectability for our attainment and for the beauty and usefulness of our manipulations. Much of this is due to professional courtesy and kindly associative action.

The praiseworthy success that has attended our unwearied efforts for improvement, is a matter of which we may well be proud.

Dentistry has kept pace with the reform movements of the age and now claims to be a profession, the peer of any other.

It has taken due and rational thought of what it should say and do, and now manifests the benefits.

With increased numbers came increased science, power and responsibilities. Once "weighed in the balance and found wanting," it has now grown corpulent and symmetrical; and can speak, act, and manfully justify itself.

In our untiring efforts to raise our profession to its present dignified and exalted position, we have done nobly for ourselves and for society.

Our object has been, and I trust will continue to be, to attain to the greatest usefulness and perfection in our indispensable vocation. Let us be governed by the exalted principles of truth, right and justice, and so rise above all narrow-minded selfishness to the lofty consideration of universal good and benefit. It has come to be thought that the Dental is an indispensable profession, for no man or woman can be a "glorification of God," without beautiful teeth, and a healthy digestion, to which these so greatly contribute.

Then it follows as a logical sequence that we work diligently for the glorification of both God and humanity by contributing so extensively toward sustaining a beautiful and dignified humanhood.

Is it possible for us then, in view of our weighty responsibilities, to rest and sleep quietly unless we are fully competent to our task.

All human souls must have their trials, losses, crosses, woes and calamities, and each one must judge of its own faithfulness or short comings, and so measure its own joys and sorrows.

All Societies, Lyceums, Associations, and the like, are composed of individuals numerous enough for the particular purposes of their association. Where the population is too sparse for the purpose such organizations are seldom met with, and the want of society is often a serious inconvenience, to say nothing of the damages occurring thereby. In cities thickly settled towns, civil, scientific, religious, social, and other organizations, are more easily formed and sustained.

In such places, these are considered indispensably necessary for securing and perfecting our inalienable rights, namely, "life, liberty, and the pursuit of happiness," by diffusing knowledge and instigating the people to the acquisition of the science of life, the most important result to arrive at, by obtaining a knowledge of the physical sciences.

Without a knowledge of these, and their legitimate results but little satisfactory progress can be made.

The members of any profession, too widely scattered, find it difficult to keep up with the discoveries of science and so obtain that skill in workmanship which renders them confident of their abilities for success, and to meet on even terms with their more favored brethern. Isolated practitioners are apt to fall into methods, manners and customs of their own which are liable to imperfections, recognizing no code of ethics or practice, being mostly a law unto themselves, and no one may without due ceremony, question their lack of attainments, or fail to commend them for their lack of judgment. But as the demand for professional men increases, these evils will, in some measure, be obviated more and more, the benefits of association will be perceived, and the members will not only profit themselves, but be enabled to render more and greater benefits to the community at large.

The dentist should be a man of nerve, skill, sound judgment and honesty, for he is called upon to relieve excruciating tortures by severe remedies, as well as to repair calamities, defects and losses.

Fifty years ago the dentist might be a cunning man, and a genius, but he could perform little or no commendable service.

His professional merit looks to us at this time of rather doubtful quality.

Such primitive line of operations is still pursued in many parts of our country, as brazen-faced as ever, and as defiant of reason and the best of common sense. In proof of this we need only consult the untruthful advertisements scattered through the length and breadth of our extensive country. But such operators sustain about the same relation to the true dentist, as did the barbers of "ye olden tyme" when they added to their vocation that of tooth drawing and plebotomy, to the most accomplished surgeon of modern times. We hope to see science and skill remove all quackery and all bungling operators from our borders, and bring out instead all the capabilities of our profession.

Noble exceptions to the primitive type of operators have here and there been found whose works, words and worship, were an honor to them and their profession. They were compensations for great quantities of delinquencies and inefficiencies.

Such men are worthy pioneers and shining lights, and are honors to their profession. They are capital media for the transmission and diffusion of correct principles. They inaugurated Dental Associations, and set the car of progress in motion, for which laudable work we have to honor and respect them.

Among the earliest organizations in our profession, and I believe the first claiming to be national in character and purpose, was the American Society of Dental Surgeons. Its constitution and by-laws were such as in the judgment of its founders, would in the best manner secure the great object in view so desirable of attainment.

Yet they were of necessity less expansive and far-reaching than the same men would institute and promulgate to-day. So it ever is in all initiatory steps taken in such directions. The old "Blue Laws" of Connecticut were considered well adapted to their time and place. Perhaps they were; but it is difficult for us now to imagine any such time or place on this continent or any other.

The mandates and required pledges of the society in question, were intended to correct certain practices and abuses which were thought pernicious, and threatened to become too widely prevalent. No society of our profession was ever organized by better men, nor men influenced by purer motives than were those of whom I would make honorable mention. Yet it was intolerant of opinion, which was its apparent fatal mistake, expressed in over-abundant legislation, which is not education, nor can it, if sumptuary in character, entail any advantage upon the members of scientific or other associations of men, for the consent of the governed is an important element for consideration in the art of governing. These remarks will apply to other and later times, yet not, I hope, with equal force.

The American Society was necessarily aggressive as all societies claiming to be reformatory must be. It was a good ship, designed for deep water and long voyages. Manned with a hardy crew and supplied with suitable armament, proudly she ploughed the muddy waters of ignoranc and empiricism. All would have been well had not the over-confident crew in an evil hour, and in the teeth of adverse gales, attempted to double cape Prejudice where she run aground on Amalgam shoals and became a total wreck, the waves of 1856 closing over the gallant craft forever. Most of the crew, "big guns," and logbook were fortunately saved. I leave the American Society to the memories of its friends with the hope that its virtues may be emulated and its failings if any it had may serve as an admonition.

The progressive minds of our specialty were true to their original purposes, and foreseeing the dissolution of the Society to which I have just referred, resolved not to abandon their efforts for the improvement of Dental Science and Art, and accordingly in July 1855 united in a call for a convention of the dentists throughout the Union to meet in Philadelphia on the 2d day of August of the same year at which time and place the American Dental Convention was duly organized upon a plan sufficiently liberal in its conception and free from that spirit of dictation which unfortunately had crept into the American Society.

This convention is still in existence and meets annually at such times and places as at each session may by vote be appointed. To its ministration I leave the Convention, and trust its founders and members will secure an adequate reward in the reflection that *some* good has been the result of its life and conduct. Closely following tne formation of the "Convention" a desire became manifest for an organization upon a basis promisory of greater advantages and benefits both as regards perpetuity and usefulness than either of the bodies to which allusion has been made.

Therefore on the third of August, 1859, persuant to a memorial previously published, some twenty-six "good men and true," representing eight local societies and two Dental Colleges assembled here on ground hallowed and made fitting by the God of Nature for good and great deeds, and proceded, pro-forma, to inaugurate the American Dental Association into the family of progressive institutions.

A provisional organization was at once accomplished, Dr. W. W. Allport being called to the chair and Dr. J. Taft was chosen secretary, which office he has acceptably filled ever since. No commendatory expressions of mine can add aught to the high estimation in which both these gentlemen are justly held by the profession for their attainments, their sterling qualities of mind and zealous devotion to the cause of progression in our specialty. Gratifying as were the auspices under which our association was established, a comparison with our present status ought to fill our heart with just pride as we contemplate the fact that we now embrace representatives from no less than nine Dental Colleges and some thirty-five local societies, the latter with largely increased representation, and with all an undiminished professional zeal. At the preliminary meeting, to which allusion has been made, a Constitution modeled upon that of the American Medical Association was reported by a committee appointed for that purpose, but was recommitted "with instructions to mature and amend, if necessary, and publish in the Journals as early as the then "next November," for as is supposed the information of the members and all other interested.

After the appointment of several essayists for the next meeting, the Association adjourned to meet in the City of Washington on the last Tuesday of July, 1860.

The Washington meeting was held in the Smithsonian Institute and so far as I can learn the procedings were eminently harmonious. During the afternoon of the first day of this session a Constitution, reported by the committee selected at Niagara was adopted, and the day following officers were selected under the same, Dr. Wm. H. Atkinson being chosen president.

Immediately after the election on motion of Dr. Fitch, the resolution adopting the Constitution was reconsidered, for the purpose of introducing an amendment to Article V. which relates to officers and their duties.

The amendment prevailed, but I am not aware that the Constitution as amended was adopted either at that or any subsequent meeting. I would here respectfully suggest that the organic law of any literary or scientific body should be concise, so expressed as to be easily understood, and be subject to amendment, repeal, or open violation only upon grounds sufficiently grave and important to justify such action. Legislation is a growing evil, and the less we as an Association have to do with it, the more shall we promote the great objects for which we join hands and maintain our assemblies. A code of By-Laws for the Association was here adopted and twenty-three names were then enrolled as members, being three less than were in attendance at Niagara the previous year.

After the adoption of several resolutions expressive of the advantage and necessity of a Collegiate Dental education, and advising "a higher order of general literary and scientific education" as requisite to matriculation in Dental Colleges, the Washington meeting adjourned to meet in the City of Cleveland, Ohio, on the last Tuesday in July, 1861. In consequence however, of the disturbed condition of the country at that time the President, by and with the advice of prominent members, very properly postponed the meeting in Cleveland to the year 1862 of which action due notice was given. Of this meeting, fortunately for your patience, I have no data upon which to predicate remarks further than to say that Dr. Geo. Watt was chosen President, and that it adjourned to meet in Philadelphia in 1863— at which time and place the session was more successful, in point of numbers at least than were any previous; but of this meeting I am again without data, yet I indulge the fond hope that my life may be spared long enough to see the full transactions from the first meeting down to the present, published and delivered to our members so that all who desire may learn more definitely of our life and experiences. Dr. W. H. Allen was chosen President at Philadelphia, and some changes were proposed in the constitution respecting the number and mode of selecting the nominating committee, (which changes were adopted at the next session,) after which, and the consideration of the usual business, this place was again selected to welcome our convocation by the majestic cadence of its mighty torrents whose constant flow admonishes us that we too are passing on.

Our Association had now passed through its experimental days, and stood, so far as dental science and art are concerned, where it now stands, the pride of its members and the hope of the nation.

Its vigorous branches now overspread the land and thousands find shelter, shade and protection beneath from the siroccos of empiricism that else would sweep, as with a besom to destruction all that is worth maintaining of our noble calling. Material aid in defining the boundaries of our profession has lately been rendered by the Legislative Assembly of the State of Ohio in the passage of an act entitled an Act to regulate the practice of Dentistry. By the provisions of this act no one can commence the practice of Dentistry, for compensation, in the State of Ohio, unless endorsed by a diploma from the faculty of some regular and duly incorporated Dental College, or a certificate of qualification from the State Dental Society or some of its auxillaries, without being regarded as guilty of a misdemeanor, and be liable to a penal assessment for the benefit of Common Schools, of not less than fifty, nor more than two hundred dollars. The hand-writing on the wall, "Jan. 1, 1873' appears sufficiently facile of interpretation that those now in practice in that State can scarcely fail to understand and act not only wisely but manfully, in converting a compliance with the law into a pleasureable duty. All praise to Ohio. God bless her and all within her borders for this advance movement so long needed—and which will I hope be repeated in every State in the Union. I award the palm to Ohio for having made the greatest improvement in Dentistry, and for publishing the best Prize Essay on our specialty extant.

New York has put her shoulder to the Juggernaut of progress and it is to be hoped that all available instrumentalities, legal and educational, will be brought into requisition to crush out of existence all quackery and imposition, that our land may blossom in the sunshine of science, and bring forth fruits worthy of our enlightened age.

Mr. President—the present is, far excellent, an age of progress, and highest and most important scientific discoveries. Its faith is not the faith that through the foregone ages was satisfactory and thought sufficient.

It has eyes and ears, and trusts in the evidence of its senses. It is an age of illumination and great events,—it lays manly hold on things now that were once only dreamed of and hoped for. Who now can fail to consider it a glorious privilege to be born for and live in such an ephoc, and to enjoy communion with its deep thought, and its most developed teachers? This is an age compulsive of deep thought and earnest reflections. The grandest results of truths, many of them long ago promulgated to the dull senses of misunderstanding are now clearly seen, understood and acknowedged on overy side. How to understand and obtain the benefits of these, should deeply concern every soul.

All causes may now be freely and fearlessly probed to the core, by suitable instruments, guided by sufficient intellect and reason. This is the age long waited for, yet sure to come, of this prophets have foretold, and for this humanitarians have waited, and in it they are now rejoicing.

A man is now just what he can prove himself to be, and mere pretentions and professions can no longer be sanctioned and recommended. All things are up for scrutiny and close examination. No closed questions now to shape ourselves to, all are laid open for investigation—are fearlessly challenged, and required to render true accounts of themselves, by serious and inevitable questioning.

We are living, we are dwelling
In a great eventful time,
In an age on ages telling
To be living is sublime.

That the marvelousness of the age may be transferred to our understandings that our own experiences in life, professionally and otherwise, may temper our conduct and stimulate us to greater attainments and usefulness—and thus render us better and happier here and hereafter is my most sincere wish and personal aspiration."

The Treasurers report was then read and adopted. The receipts for the past year were $975. and the expenses $884.

A resolution authorizing the revision of the roll of membership was passed.

Dr. McKellops made a motion, which was adopted, to suspend the rule for the purpose of giving Dr. Westcott an opportunity of explaining the process of a recent remarkable case of teeth-regulation which had come under his practice. He said the first effort to move a tooth should be very slight, for then more could be done at the second effort with less pain. Neither springs nor elastic substances should be used if it were possible to avoid it; and to the almost exclusive use of wooden wedges, in the present case, did he attribute the absence of inflammation, notwithstanding the great extent of the movements attained. He preferred that the plate should be used in the mouth; but where that was unavoidable, it should be dispensed with at the earliest possible moment. He made it a rule to obtain the lateral movement first, and finish that, before beginning the longitudinal movements. Where the teeth are so short as to make it difficult to get hold of them, he drills into them and inserts a gold screw or staple by which to make attachments. Any ill effect of any regulating apparatus, upon the substance of the teeth, might be obviated by removing the plate and cleaning it after each meal, cleansing the teeth and rinsing the mouth with soda water. The speaker exhibited all the apparatus used in this case which was very elaborate and ingenious, but which could only be described at great length and by the aid of engravings. The process of regulating was a very tedious one, occupying several months; but the result was the perfect arrangement of what had been an extensive malposition of the teeth of the upper and under jaws.

Dr. Shepard made a statement in regard to the distribution of the printed proceedings of this body for the years 1865 and 1866.

Dr. Sage offered a resolution inviting the members of the press now in this place to visit the Association while in session. Passed.

Dr. Whitney submitted a resolution welcoming the members of the profession from Canada as members of this Association. Dr. Westcott suggested an inquiry relative to the laws of Canada as affecting dental practitioners in that country from this side of the line.

Leave was granted to Dr. Scott, of Canada, to explain the Canadian laws relating to American dentists. The gist of the statement made was, that naturalization (which requires a residence of two years) is necessary before an American dentist can practice in the new British Confederacy.

Dr. Westcott moved to amend by simply inviting the Canadian bretheren to seats on this floor.

Dr. Shepard made a point of order on the resolution. The resolution was declared not in order, and Dr. Westcott's amendment as a resolution was adopted.

Drs. McQuillen, Horne and Allport were appointed a committee to ascertain whether those presenting credentials were all practicing dentists. Adjourned until 4 P. M.

AFTERNOON SESSION.

Met as per adjournment and called to order by President Taft, when the minutes of the morning session were read and approved.

Accepted report by Dr. Bogue, of the Auditing Committee.

Dr. Wetherbee moved that the Association now proceed to determine the place of the next meeting of this body.

On proceding to ballot, Saratoga was selected.

The committee on Credentials reported that informalities have been admitted in the organization of this body at the present session; which report also included the following form of certificate for the use of members hereafter:

This certifies that———was duly appointed a delegate to the American Dental Association, from the Dental Society of ———, on the———day of———, 18—, and that the said——— is a dentist of good character and standing, and at this time in regular practice.

Signed, ——— ———

Secretary of the Dental Society of ———.

As much excitement ensued as on the previous day and after much discussion Dr. Cheesebrough called for a division of the question. After further debate, (regular and irregular) Dr. Horne re-read the report in parts, all of which was rejected with the exception of the form of certificate, for the adoption of which there was a large affirmative vote.

Dr. Atkinson moved the appointment of a special committee for the revision of the Constitution and By-laws; passed.

Adjourned to 10 o'clock, Thursday.

THE THIRD DAY'S SESSION.

On motion of Dr. J. Allen an opportunity was given to Dr. Kingslep to explain his method of supplying artificial palates. Dr. K.'s time was limited; but he seemed to improve it to the satisfaction of his hearers, and a vote of thanks was passed for his clear elucidation of the principles and process involved in the specialty named.

Dr. Kingsley remarked that two years ago, at the meeting in Boston, he had given the result of his experience in full up to that time, but that since then he had made such advances in simplifying his appliances as to render success easy to any dentist of moderate skill. He desired his hearers to keep clearly in mind the distinction between congenital and accidental deformities. Patients suffering from the former require months, and sometimes years, to overcome the difficulties of articulation, even with the best-fitting appliance; while in accidental lesions, not only is the appliance much more simple in its character, but the results are attained immediately on its introduction. He was very particular that this discrimination should be made, in reading the reports of cases published from time to time, as it was not unfrequently the case that great claims were put forward, for so-called improvements, when a closer scrutiny or inquiry would reveal that it was a case of accidental deformity, and the results attained were no indication whatever of what would be done in congenital defects. He instanced the case of a patient, a man nearly fifty years of age, for whom he had made an artificial palate within the last month, where the natural soft palate was entirely gone, and whose speech could not possibly be understood by any stranger; and yet immediately on the introduction of the new palate, his speech was entirely restored. This defect was the result of disease, and although it had occurred nearly thirty years before, and during all that time his power of articulation was gone, his restoration, on the application of the palate, was complete. If the deformity had been congenital, although it might have been precisely the same in locality and extent, pure articulation, at that age, would probably never have followed.

He presented an impression in plaster which embraced the teeth, hard and soft palates, together with the nasal cavity and the chamber of the pharynx, and briefly described the method of manipulating, which was substantially the same as that published in the previous reports of the Association. His improvements, within the last two years, were in the form of the palate, and the mould in which it was made.

The mould, while still of type metal, is so simplified that instead of being, as formerly, in four pieces, it is now made in a flask of two pieces, with a brief outlay of time and skill. By placing a ring, of the same size, between the halves of the flask, thus separating the type metal dies, and filling the space with a more fusible alloy, the mould is doubled, and the process can be repeated indefinitely, where a number of similar instruments may be required—the palate produced from such a mould being the same in form and utility as that made from the mould of four pieces.

Another improvement was a light framework of gold, provided with a joint, the frame to support the rubber flaps or wings for an artificial palate, and the joint to permit the perpendicular movement necessary to correspond with a similar movement of the natural palate. This method of making an artificial palate is such that the same frame and rubber wings can be applied to nearly all cases of fissure which do not extend beyond the soft palate, and in an emergency, the rubber wings need not be made expressly for it, but may be cut out of the sheet rubber of the market. He exhibited his instruments, also his moulds, describing the various processes of manipulating; but a more detailed description than the foregoing would necessitate engraving for its comprehension. A unanimous vote of thanks was passed by the Association.

The report of the standing committees being now called, the first in order was that on Dental Pathology and Surgery, which was presented by Dr. Atkinson.

Dr. Bogue from the same committee, read a paper recapitulating the points which, in this department, had attracted special attention during the year. The question of the dentinal tubuli; of the effort of the dental pulp to protect itself from the approach of decay by the consolidation of the intervening dentinal tissue; and the result of recent effort for the preservation of the exposed pulp of decayed teeth, with other points of less importance, were commented on and afforded texts for discussion. Admitting that only a careful record of a large number of cases can be relied on to prove any theory correct, he inferred, though he could not consider it proved, that the pulp is as capable of reparative processes as other vascular structures. Salivary calculus was treated as a direct source of injury, and its perfect removal necessary, to be followed by an application of escharotics to induce renewed action of the peridental membrane. The treatment of epulis was touched upon. An apparent extripation was not deemed to be sufficient, but a removal of the surrounding healthy tissue was demanded, to be followed by thorough cauterization.

Dr. J. S. Dodge, Jr. referred to certain dark-colored nodules found upon roots of the teeth, and even upon their extreme ends. He could not agree with the common opinion which considered these to be deposits of a calcareous nature from the saliva; there was no practical limit to the deposit of tartar upon the exposed portions of the tooth, but it was confined to the border of the gums, and was of a light color. Black nodular deposits were never found but on the roots of teeth, under the soft part, and never in large masses, and their tendency was to work to the apex of the fang. Some other source must be found for them than that of deposits from the saliva. He had tried to make microscopical sections of them, which, from their size and formation, was difficult, but in two instance he had partially succeeded. The whole mass was composed of very regular concentric layers of accumulation, in aggregated spheres, not similar to any organic form in the body. So much had been seen: his conclusion was, that these nodular masses were deposits of calcareous matter similar to those thrown out by the periosteum for the formation of bone, but being in a diseased condition, the process was merely carried so far as to form aggregations on the surface.

Dr. Atkinson had never seen such a deposit, except where it had advanced, as tartar does, on the recession of the gums; and even where the gum and peridontium were disconnected from the tooth to such a degree as to loosen it, he would not dispair of restoring the original attachment. His process was to thoroughly remove every portion of the deposit, whatever its character, not being at all fearful of cutting into the substance of the cementnm, wash clean, and inject once thoroughly, with a hyperdermic syringe, a solution of chloride of zinc. This treatment he would pursue, not merely for nodular deposits, but also for rings below the margin of the gums. He remarked that it was questionable whether hard tissues once fully formed undergo any changc.

Dr. Butler inquired how Dr. A. got at the end of the root for deposits at that point.

Dr. Atkinson replied that he had followed up the open channel, whether by the side of the tooth or a fistulous opening through the alveolus, and rasped of the end of the root, never disturbing the connections between the soft parts and the tooth, at its neck, when that was intact.

Dr. Butler had never seen the accumulations spoken of on roots where the periosteum was in intact, but found it common on the parts denuded. When he deemed amputation admissable he would have no fear of disturbing the alveolus, unless the condition of the patient forbade any operation whatever, but would clip off the apex of the root in whatever manner was most convenient.

Dr: Bogue alluded to cases coming under his observation directly from other practitioners, who pronounced the teeth free from tartar; yet the teeth would be lost if left as they were. The only indications in these instances was a redness of the margin of the gums, and he found always that the alveolus was absorbed and the periosteum detached. He had never practiced the amputation of the end of the root, but had succeeded in removing the nodules after repeated effort, and then made mildly escharotic applications.

Dr. C. Palmer had never found any depositions on the roots, except where there had been external openings, and believed the one was necessary to the other.

Dr. W. H. Morgan was not prepared to concede that these deposits were tartar; their nature was still a question in his mind. He recalled a case in which there was a considerable deposits of a calcerous nature on the root of a tooth, with no external opening, in the vicinity of which there had been a tumor.

Dr. H. Judd said it must be conceded that the origin of these masses is either from inflammatory action or by deposit from the saliva. In the latter case there must be external communication, and in recalling instances to mind they supported this hypothesis. Carbonate of lime, when mixed with fatty substances, form in globular masses.

Dr. Allport in looking back, could not remember having seen a case of black deposit on the root, where the gum and alveolus were intact at the neck of the tooth. Other globules, of much lighter color, found under the soft parts, without an external opening, were doubtless the result of diseased action, as suggested by Dr. Dodge. There being no means of escape for the secretion under these conditions, it aggregates upon the surface of the root.

Dr. McClelland was of the opinion that deposits from the saliva would be found where that secretion was most abundant; its being found under the gum was conclusive evidence to him that it was thrown out by the peridental membrane.

Dr. McQuillen agreed with the opinion that tartar could not be deposited without an external opening. Referring to the intimation of a doubt whether the hard tissue could undergo change, he said there was no tissue in the animal or vegetable kingdom, with the possible exception of the enamel, which does not undergo continual change. Have not the investigations and experiments of Hunter, Duhamel, and others proved this most conclusively? What are our senses worth, if we do not use them to observe the phenomena everywhere presented. Time could be more profitably spent in observing, recording, and generalizing facts than in getting up fanciful theories, expressed in language incomhensible to ordinary minds. Dr. Lionel S. Beale was the first to take the exception to the general view relative to changes in hard tissues, and the speaker combatted his statement several years ago. He cited, in proof of constant change, the formation of the frontal, sphenoidal, and maxillary sinuses, the diploe in the flat bones, and medullary canals in the long bones, and the process by which the deciduous teeth, after being built up cell by cell, at the proper time, by a retrograde metamorphosis, are absorbed cell by cell. In the permanent teeth the evidences of change were constantly occurring—as in hypertrophy of the cementum, and the not infrequent absorption of the cementum and dentine in other cases. Permanent teeth extremely soft in early life become almost as hard as flint at a more advanced age. Again, teeth which had been quite dense and perfect in their structure, as in the case of females, after the commencement of the maternal functions lose much of their former hardness, owing to the waste constantly going on not being supplied by a sufficient amount of material to meet the demands of mother and child; in which case the latter is nourished at the expense of the former, and the mother's bones and teeth become softened. He drew attention to these facts mainly to induce greater efforts for the preservation of the teeth of young persons, as operations, the success of which might almost be despaired of, would frequently prove of lasting benefit, owing to an improvement in the texture of the dental organs.

Dr. John Allen urged the necessity of providing in the food materials which should supply, atom by atom, the wants of the system. He asserted that, as a nation, Americans have the worst teeth of any people, because they change the proper proportions of the constituents of the food; while in countries where this is not done, the people retain their teeth, sound and beautiful, to old age. To show the gigantic scale on which we do this, he said that from every barrel of superfine flour, 40 pounds of mineral matter were rejected in the process of manufacture; and, allowing half a barrel of flour as the yearly allowance of a child, every child is deprived of 20 pounds per annum of the material most necessary for perfecting the osseous structure of the body, making an aggregate loss to the system, in twenty years, of 400 pounds. To accomplish this great waste, 28,000 men are employed, at an outlay of $9,000,000, and a destruction of 20,000,000 pounds of the material essential to the formation of sound teeth and bones. To offset this evil, 10,000 dentists are employed by the American people, but their efforts are entirely inadequate to compete with the destructive agencies above named. When the conclusion is accepted that proper diet is the highroad to improvement in the quality of the teeth, he would have hopes of a stronger and healthier organism; but any means short of a radical change in this respect would prove inadequate.

Dr. Atkinson said the whole philosophy of filling teeth depended on the assumption of the durability of their structure. He delights in putting things into the teeth that irritate them. Without irritation there could be no nourishment; the similarity or dissimilarity of the irritation, produced by remedies or food, to that required by the organism determined the ascendency of health or disease. The rule had been that when the pulp of a tooth became exposed it should be devitalized and removed, and the root filled to its end. He asserted that a man was weak or wicked who would willfully destroy a tooth-pulp; all that was necessary was to use an agent which would coagulate the albuminous surface of the exposed pulp, and the subsequent operations were certain to be successful. His practice was to cover the exposed pulp with creasote, upon which he dropped oxychloride of zinc in a plastic condition; when this had hardened, he proceeded to remove the excess of the material and fill the cavity as usual with gold. If the inflammation had gone on to suppuration, he would fill temporarily. Suppuration is the production of pus, and pus is a fluid composed of serum and white corpuscles deprived of their vitality. All the tissues of the body are made up of corpuscles, white forming the nerve tissues; the red, the muscles. Cohnheim has seen the corpuscles pass through the walls of the capillaries.

Dr. B. T. Spellman said that he did not understand the white corpuscles to be component of pus, but that their character was changed by diseased contact. He had heard, from many witnesses, that the treatment of exposed pulps just detailed was in their experience productive of much pain, and therefore he considered it bad practice.

Dr. McQuillen said pus was regarded, by the best authority in surgery as a fluid, composed of dead exudation corpuscles floating in serum, these exudation cells, in their normal condition, being the tissue builders. It was impossible that white corpuscles could pass through the walls of vessels to be incorporated in the structure of nerve tissues. He had been accustomed for years to demonstrate to his students the circulation of the blood in the frog's foot, tongue, lungs, and in the mesentery, and had watched the process by hours, and had yet to see the phenomenon referred to. Admitting Cohnheim's observations to be correct (which he was not disposed to do,) the propriety of drawing deductions from pathology to sustain physiological theories was a very defective and illogical method of reasoning. The number of persons who had seen ultimate nerve fibres was exceedingly limited. Only those who had employed the higher magnifying powers could lay claim to this distinction. The corpuscles rolling through the capillaries might appear to pass through the sides of the vessels, but it was only an ocular deception. The calibre of an ultimate nerve fibre was infinitesimally smaller than that of white corpuscles, and, in addition, it was made up of three distinct structures, the neurilemma, the white substance of Schwann, aud the axis cylinder. How, then, can a corpuscle make up such a nerve fibre? The same objections held good with respect to muscles being formed from the red corpuscles. The ultimate muscular fibrillæ (which consists of myolemma, a membranous sheath inclosing the sarcous elements) is much more minute than the red particles of the blood. Many years ago similar views to those presented here were advanced by Doellenger and Dutrochet, and their fallacy was then exposed. The perfection at which microscopy has arrived, and the use of good instruments, enable us, by comparing with precision the size of different parts, to completely refute this untenable theory.

Dr. Wetherbee stated that, two years ago, when he spoke of the use of the oxychloride for capping pulps, Dr. Atkinson expressed his doubt of its adaptability. He gave his experience in a number of cases where he had succeeded in its use. Frequently there was pain for a short time, but without subsequeut uneasiness. He does not consider the pain dangerous, or the material in any manner endangering to the pulp; it acts as an astringent. Two cases in which the pulps bled, two years ago, now have all the indications of living structures.

Dr. J. S. Dodge, Jr., said, if the effort to preserve exposed pulps were any new thing that had not been tried heretofore, he would go home to try it with a good deal of zeal. But this was not the case; it had been a favorite practice years ago with the old practitioners, and when he commenced his practice he was somewhat enthusiastic about it, though even then the old men had begun to shake their heads about it, and since then they have been shaking them harder and harder, until the operation of filling over exposed dental pulps had gone out of date. Now a new material was coming into fashion, it appeared for the same purpose, oxychloride of zinc. Put this in a sensitive tooth, and it would cause severe pain, and its effects upon an exposed pulp, he believed, would be ultimately to destroy it.

Dr. Allport said that exactly what proportion of exposed pulps could be preserved by any method could only be stated after the comparison of a large number of recorded cases. After the explosion of the theories of Harris, those who enunciated any method of saving pulps were looked at with a quizzical glance. He then referred to the operation which he had introduced, consisting of an excision of a portion of the exposed pulp, which was relieved of congestion by the consequent bleeding, leaving flaps to come together and heal by first intention—admittedly a very difficult operation—and claimed that he had by that method saved a large number of teeth, and obtained a new calcareous deposit at the point of exposure.

The treatment prescribed by Dr. Harris gave the pulp the best possible chance to inflame and suppurate, by leaving a space between it and the cap. He would have gentlemen not deny what they had not tried : he had not used the oxychloride, and knew nothing about it, but half believed what had been said about it to be true. He believed there was something living in the pulp. Kölliker, years since, said the dentinal tube or canal was an elongation of the dentinal cell resting on the pulp. In a recent work he reiterates this, and says this is the channel through which the calcareous mass is deposited, and this would account for the increased density of portions of dentine near points of decay.

Dr. Wetherbee, in answer to a question, qualified his previous statements as referring to pulps simply exposed, not inflamed. He said that no large amount of oxychloride should be allowed to remain in the tooth longer than a few weeks, as the free acid in the preparation would act most injuriously upon the substance of the tooth.

Dr. A. W. Freeman said he had obtained as good results as that with Hill's stopping.

Dr. Kennicott thought misunderstanding arose from some attributing a medicinal virtue to the material under consideration, whereas he considered any good resulting from its use to be due to mechanical causes. No sensible man would proceed to fill permanently over an exposed pulp until he was sure that it was in a healthy condition; every application previous to that should be considered preliminary treatment. The oxychloride was a substance which, applied in a plastic condition, adapted itself without pressure to the exposed vascular pulp, and then on hardening, protected it for a time from the action of external agents.

Dr. Horne said he had waited up to this time in the expectation that the various advocates of the new plan, if let alone long enough, would destroy one another's arguments, and his expectations had been fulfilled. The use of oxychloride of zinc had been first brought to general notice at the meeting, two years ago, at Boston, where Dr. Keep, of that city, claimed large success in a number of recorded cases, It was a question in his mind if the material which Dr. Keep used was at all like the material sold under that name in the dental depots; and the gentlemen who had thus far spoken used the oxychloride of the shops, and not Dr. Keep's preparation, as far as he could judge. The whole claim really amounted to about this; A preparation, called oxychloride of zinc, consisting of a white powder, which is mixed into a mortar with an acid fluid, is plastered into the tooth over an exposed pulp; the patient has more or less pain for a longer or shorter time, and then it stops; after hardening, cut away the surplus material and fill the cavity with gold, and, where there is no subsequent pain, conclude that the pulp of the tooth is alive and all going well. Some modify this process by covering the exposed pulp with creosote before putting in the mortar, and claim that there is then no pain felt. A great many who had tried the same process had had a great deal of subsequent pain to contend with. But all this superstructure had been built upon the slight foundation of some temporary apparent success, opposed by a great deal, perhaps more, of evident failure, in the face of the well-known fact that teeth containing dead pulps might lie dormant for years and then break out into the most troublesome activity; and also that pulps, after having been exposed, might on the condition of exclusion of air and moisture, quietly die and become atrophied. On the other hand, it was as certainly true that an exposed pulp was occassonally found which, having maintained its healthy vitality, and being protected from external irritation, threw out from its enveloping membrane a deposit of secondary dentine, which more or less perfectly shuts up the opening into the pulp cavity. But these cases were so rare that men would go from one city to another to see them. Admitting all the favorable cases cited to-day as perfectly true, so far they were utterly insufficient to prove that the pulps in question were not now dead, or undergoing a slow destruction by the free acid whose presence had been so incautiously alluded to, or by the powerful escharotic (creosote) used to abate this pain caused by the acid, and whose legitimate action would be to destroy the soft tissue with which it came in contact, insuring to it only a less painful death. While experimentation was to be sedulously encouraged, he deprecated the confident assertion, as fact, of what was only supposition, Let us see what a few years may bring forth. Other theories, as well supported as the one here presented, had needed but a short time to run themselves out; and he was therefore the more careful not to fall at once into every new current, but rather disposed to prove all things.

Dr. Hitchcock was glad that oxychloride had now so many friends; two years ago, in Boston it could hardly get a hearing. He refered to a number of cases that he had reported to the Massachusetts Dental Society during 1866–7, and published in the dental journals. A majority of all these cases he had seen since, and on examination by means of cold applications, found the pulps alive. The material for sale in the dental depots was not the same as that used by Dr. Keep and by himself; on the contrary, it contained a great deal of impure and sometimes deleterious matter; he had found that of a yellowish color better than the pure white.

A paper was read from Dr. Knapp, of New Orleans," On Hidden Dental Caries," which was referred to the committee on Publication.

The Association then adjourned to 3 o'clock P. M.

### AFTERNOON SESSION.

Met as per adjournment, when the minutes were read and approved.

Dr. Wetherbee, from special Committee, made a report in regard to compensation; and on motion, it was voted that fifty dollars each, be annually paid to the Treasurer and Recording Secretary, for their services.

An additional report from the Committee on Credentials was adopted.

Further time was granted to the chairman of the Committee on Publication to report.

The discussion was resumed on Dental Pathology and Surgery, and was continued foa some time, was generally engaged in, and seemed to elicit a very lively interest on the part of the members present, when the meeting adjourned until half past eight o'clock in the evening at Spencer Hall.

At the evening session it was voted on motion of Dr. Judd, the discussion on Pathology and Surgery be closed.

No report being made on Dental Physiology, the Committee on chemistry presented a paper from Dr. H. S. Chase on the "Character and Uses of Saliva," which was read by Dr. Wetherbee.

It stated the characteristics of this fluid, with directions how to obtain it for testing its reactions; quoted various estimates of the quantity of this secretion, and its chemical composition; gave the results of a number of the author's experiments on different animals, in which he found the saliva to be acid; and concluded that healthy saliva is not neutral in its reaction in man or in the lower animals, and that nature makes a strong effort to place the saliva in a physiological condition at the time food is being masticated. The offices of the saliva were next noticed, and some general considerations adduced as to the organic matter found in it.

After some very interesting remarks by Drs. Wetherbee, Atkinson, Kennicut, and Buckingham, the Association adjourned until Friday morning at 10 o'clock.

FOURTH DAY'S SESSION.

Met according to adjournment when the minutes of the two previous meetings were read and approved.

A resolution that the Treasurer be henceforth paid $50 per annum, for collecting the fees of members and taking care of the accounts, and voting the same amount to the Secretary, on condiiion that he prepare the Transactions for delivery to the Publishing Committee, was also adopted.

The report of the Publishing Committee of 1866 was presented by the chairman, Dr. Shepard. The total cost of the volume of 512 pages, embracing the reports of two years, 1865 and 1866, brought out by them, is $1110, for an edition of 500 copies; $600 of the amount had been paid, leaving a balance of $500 due the publisher. After the distribution of the copies to all the members of the Association entitled to them, there will be between two and three hundred copies left.

The report was accepted with the thanks of the Association. The Treasurer was instructed to pay the balance of $500 due. On the recommendation of the committee, a number of copies were donated to various learned societies, dental colleges, and dental and medical magazines, in this country and Europe. The further distribution to members and authorized sale of surplus copies at $5 each, was left in the hands of the committee.

The Committee on Nominations made the following report, which was adopted without change:

STANDING COMMITTEES FOR 1868–69.

*Committee of Arrangements*,—J. G. Ambler, New York; L. D. Rogers, Utica; L. S. Straw, Newburg.

*Committee on Dental Pathology and Surgery.*—J. S. Latimer, New York; M. DeCamp, Mansfield, Ohio; G. T. Moffatt, Boston; A. B. Robbins, Meadville, Pa.

*Committee on Dental Physiology.*—W. H. Morgan, Nashville; A. Westcott, Syracuse; H. S. Chase, St. Louis.

*Committee on Dental Chemistry.*.—T. L. Buckingham, Philadelphia; H. A. Smith, Cincinnati; A. Lawrence, Lowell.

*Committee on Dental Education.*—J. Taft, Cincinnati; F. J. S. Gorgas, Baltimore; M. S. Dean, Chicago.

*Committee on Dental Literature.*—R. W. Browne, New London; L. D. Shepard, Boston; J. McManus, Hartford.

*Committee on Dental Histology.*—J. H. McQuillen, Philadelphia; W. W. Allport, Chicago; J. S. Dodge, Jr., New York.

*Committee on Operative Dentistry.*—C. R. Butler, Cleveland; J. S. Knapp, New Orleans; W. H. Allen, New York.

*Committee on Mechanical Dentistry.*—John Allen, N. W. Kingsley, New York; C. H. Harroun, Toledo; J. A. McClelland, Louisville; S. B. Palmer, Syracuse.

*Committee on Prize Essays.*—G. H. Cushing, Chicago; A. L. Northrup, New York; W. F. Morrill, New Albany, Ind.; C. E. Francis, New York; F. N. Seabury, Providence.

*Committee on Voluntary Essays.*—M. S. Dean, Chicago: A. M. More, Lafayette, Ind.; W. P. Horton, Cleveland.

*Committee on Dental Therapeutics.*—E. A. Bogue and A. P. Merrill, New York; I. J. Wetherbee, Boston.

*Committee on Publication.*—Homer Judd, Edgar Park, E. Morrison, all of St. Louis.

*Committee on Instruments and Appliances.*—W. C. Horne, New York; Corydon Palmer, Xenia, Ohio; J. A. Bishop, New York.

The report of the Committee on Dental Education was presented by Dr. Homer Judd. He said the subject was divisible into two parts—ths first having reference particularly to the acquisition of a practical knowledge of the details of dentistry, while the other referred more particularly to that course of mental culture which marks the dividing line between the tradesman or mechanic and the professional man. The dental journals should not proceed, in their educational efforts, upon the idea that their duties consist entirely in disseminating theories and chronicling new facts; but they should keep in mind that the great want is a general dissemination of *known* facts—dental text-books, like others, being generally far behind the times when they come into the hands of the profession, and not as generally read as the journals. The standing of the profession does not depend, to any great extent, upon the knowledge of dentistry which its members may possess, but its position among the liberal professions is determined rather by the culture and literary acquirements of its members. He thought no one could maintain his position as a man of learning and science who did not daily apply himself to the studies and investigations necessary thereto. While appreciating the efforts of our dental collegiate institutions in the cause of education, he declared his conviction that the future status of the profession would depend more upon what they shall demand as prerequisites to admission to their classes, than upon the curriculum of study there pursued. Judging the progress of dental education by the number of students the past year, the conclusion would not be flattering; but, to offset this, a higher standard has been required, and the decrease in number might be attributed to general pecuniary embarrassment rather than to any disatisfaction with the course pursued by the colleges. He then remarked upon the value of diplomas as dependent upon the character of the institution from which they issued, and commended the passage of a law in Ohio, requiring the possession of a diploma as a necessary qualification to practice dentistry legally. He encouraged a wider range of education for dentists, in order that their sphere of usefulness might be enlarged, and they be placed on a level with recognized medical specialists.

The report was followed by a paper of a very general nature from S. J. Cobb, of Nashville, Tenn. presented by Dr. W. H. Morgan.

The report on Dental Literature was made by Dr. M. S. Dean. No new work, except that of Dr. Arthur on "Decay of the Teeth" had been added to our dental literature during the year. The views presented in this volume were regarded as peculiar and antiquated. The author's views, in favor of "separating all the teeth back of the eye teeth, if the incisors are decayed before the twelfth year," or even,

"if there is a reasonable certainty that the back teeth will decay, separating them before the decay occurs, as the object is then effected with less loss of the substance of the teeth," met with no favor in the report. The second edition of Dr. Taft's "Operative Dentistry" is noticed and commended for many improvements in its style; some of the author's statements are criticised, and regret expressed at the absence of any details in regard to the treatment of dental periostitis and alveolar abcess. The third edition of Dr. Harris' Dictionary, revised and enlarged by Dr. Gorgas, is very highly commended as a vast improvement on the former editions. The work is said to be as perfect as any dictionary of a science or art, running before it at so rapid a rate, can be; forming a lexicon in which can be found nearly every term belonging to the nomenclature of dentistry. Dr. Watt's "Chemical Essays" come in for hearty econiums, both in relation to the matter and mechanical dress. The beginner will find himself irresistibly drawn on in these pages to a paradise of beauty which he becomes eager to explore; and the student, at the close of his term, will find new ideas so quaintly and forcily expressed and clearly illustrated, that they will become indelibly fixed in his mind, and ever ready when their principles are required in his practice. The author is said to treat his subject in a truly poetic spirit, and in the most animated nature imaginable, while in his highest flights he does not leave his subjects trailing in the mud, like the too heavy tail of a kite, but carries it along with him clearly and beautifully, in the full sunlight of science, evolving principles with scrupulous and mathematical exactness. Attention is given to our periodical literature—the *American Journal of Dental Science*, *Dental Cosmos*, *Times* and *Register*, all being commended to the support of the profession. In regard to each journal, the report expresses the opinion that their value would be greatly enhanced if much that is but remotely allied to our specialty could be displaced by articles of a more strictly practical nature. This remark is extended in its application to all the dental journals. The report further complains, that in all our dental journals there may found articles of interest to our profession, showing considerable research on the part of the writers, which at the same time exhibit reprehensible carelessness in their preparation for publication; and then, again, a writer occasionally expends his force in his composition, and in impressing upon the minds of his readers the importance of the subject that he is *about* to treat, but which ever coquettishly eludes his grasp. The former class, while they annoy, instruct; the latter only annoy and disappoint the reader. The feeling of the profession is stated to be one of dissatisfaction with present attainments; no improvements astonish, and no discoveries startle, the supreme feeling being a longing for something yet out of reach, and for which they eagerly look to the dental journals as the *avants couriers* that herald each advancing step. The report recommends a more liberal pecuniary support as the most effectual remedy, to be heroically applied by every member of the profession, for any evil that may exist in our periodical literature.

The report on dental histology was presented by Dr. S. P Cutler. It reviewed the statements and theories of Kölliker, Virchow, Tomes, Beale, and other investigators of the structure and formation of the dental tissues, and announced the results of his own microscopical observations in the same field.

Dr. Judd took exception to the discoveries announced by Dr. Cutler, on the ground that it was a well-settled rule among scientific men that any new discoveries should be accompanied, on their announcement, by such details of the processes passed through as would enable others to go over and verify each step of the investigation. At the time that Tomes demonstrated the existence of fibrils in the dentinal tubules, the existence of ultimate nerve-fibres had not been demonstrated by Beale; but the latter investigator had since discovered that the germinal matter of the dentine penetrated the tubules, and had seen that the ultimate nerve-fibres break up and penetrate the germinal matter. The dentinal tubules, being one ten-thousandth of a line, can of course be penetrated by the ultimate nerve-fibres, which are only one hundred-thousandth; and their presence fully and satisfactory accounts for the sensitivenese of the dentine. As to the theory that the dentinal fibrils of Tomes were a post-mortem product of the coagulation of albumen, it was only necessary to recal the fact that albumen does not spontaneously coagulate.

Dr. Atkinson said he had never yet been able to see the termination ol an ultimate nerve fibril, and did not think any one else had seen it.

Dr. J. S. Latimer exhibited, under a microscope belonging to Dr. Taft, a section of a perfect adult canine tooth, showing interglobular spaces, colored with carmine, with the dentinal tubuli passing across them. He remarked that this specimen had a historical interest, as it was an additional confirmation of the position maintained by Dr. McQuillen, which had been so much controverted by others.

Owing to the great amount of business pressing, a resolution was adopted allowing members to write their views, on the subjects of the various reports, and send them to the Publication Committee.

The Association then fixed the hour of final adjournment for 6½ o'clock P. M.

### AFTERNOON SESSION.

The Association met pursuant to adjournment.

The following amendments to the constitution, proposed by Dr. B. F. Spellman in 1866, were adopted, namely:

To insert between Sections 2 and 3, of Article III., the following:

"To be entitled to representation in this Association, dental societies shall be required to adopt or substantially recognize its Code of Ethics."

And insert between Sections 4 and 5, of the same article, the following:

"Any member of a local or State association who is expelled from the same shall cease to be a member of this Association from the date of his expulsion."

An amendment, changing the time of the annual meeting from the last Tuesday in July to the first Tuesday in August, was also adopted.

The Committee on Operative Dentistry made no report.

The Committee on Mechanical Dentistry reported, through its chairman, Dr. B. T. Spellman. He noticed, in order, the several materials in use as bases for artificial teeth As to Colburn's material, the report agreed with the manufacturer that it would answer for temporary use, not to exceed a few months. Newbrough's rubber being prepared by a new process, the committee are unable to report whether it will stand the fluids of the mouth or not. It possesses the same properties as dried vegetable ivory, and that soon softens in the mouth. The wicked raid made by the rubber company had induced some good men to be too sanguine of it. The Simpson rubber has disappointed the profession. Dr. McClelland was invited to come before the committee and explain his process, but he neither came nor replied. Of the merits of this material they know nothing, but are glad to report that there is much talent and industry devoted to the discovery of something which will take the place of rubber. The porcelain base is recommended as growing in favor and unsurpassed for cleanliness ; the contour of the face is as well restored by it as by the platina and continuous gum work. There is no trouble in making an upper or under set ; it can be ground to fit the the plaster cast in about the same time needed to get up a set of rubber blocks ; the marks of the stone to be obliterated by a coating of gum enamel fused on afterward.

Artificial palates have been simplified to such an extent as almost to render staphyloraphy obsolete.

The committee reported no improvement in the status of mechanical dentistry, the advent of rubber having driven the best men from the laboratory in disgust. They recommended only gold for partial cases, and Dr. J. Allen's continuous gum as the ideal fully attained in supplying lost teeth and restoring the contour of the face.

Dr. A. W. French on behalf of the Committee on Prize Essays, reported that no essays had been presented, and the committee was inclined to think its title a misnomer. The Chairman, Dr. French, presented an essay on the necessary professional qualifications for the practice of dentistry. He did not favor the passage of restrictive laws; he doubted their agreement with the institutions of the country; he would rather rely on the profession protecting itself by suitable ethical regulations in the local societies.

Dr. Palmer from the Committee on Voluntary Essays, read a paper on taking partial impressions.

Dr. C. R. Butler, from the Committee on Dental Therapeutics, reported that he had seen the best results, in treating sensitive dentine, follow the use of carbolic acid and the acetate of morphia; he did not consider it safe, however, under all circumstances. He had found tincture of aconite root to work favorably in cases of facial neuralgia or peridontitis. Chloride of zinc, in varied dilutions, is a valued remedy in the treatment of many oral diseases. He did not perceive the advantage of phosphate of lime to remedy defective dental tissues; he believed there is not a lack of material but of assimilative power in the organism.

A special committee, of which Dr. Kennicott was chairman, on neuralgic affections, was given further time, with instructions to report at the next session.

A special committee on Instruments and Appliances, consisting of Drs. Shepard Bogue and Smith reported improvements in dental chairs, by Drs. J. B. Morrison, O. C. White, I. A. Salmon, and W. M. Butler; a pneumatic mallet, by B. Bannister; an automatic mallet, by I. A. Salmon; hand pressure and mallet pluggers, by S. C. Taylor; an improved regulator for controlling the heat in making nitrous oxide gas, by F. Searle; an automatic apparatus for the same purpose, by A. W. Sprague; an improved pin for artificial teeth, by J. A. Mason; pluggers, excavators, nerve instruments, etc., of improved patterns, by S. S. White; extension bracket, with gas annealing lamp combined, by Buffalo Dental Manufacturing Company; a compressor for closing flasks inside the vulcanizer, by G. Hays; a porous duct compressor, by A. P. Southwick.

The time of the session having now expired by limitation, the Association adjourned to meet at Saratoga, on the first Tuesday in July, 1869.

---

